# Antibody Testing in Estimating Past Exposure to *Chlamydia trachomatis* in The Netherlands Chlamydia Cohort Study

**DOI:** 10.3390/microorganisms7100442

**Published:** 2019-10-11

**Authors:** Bernice M. Hoenderboom, Michelle E. van Willige, Jolande A. Land, Jolein Pleijster, Hannelore M. Götz, Jan E. A. M. van Bergen, Nicole H. T. M. Dukers-Muijrers, Christian J. P. A. Hoebe, Birgit H. B. van Benthem, Servaas A. Morré

**Affiliations:** 1 Centre for Infectious Disease Control, National Institute for Public Health and the Environment, Antonie van Leeuwenhoeklaan 9, 3721 MA Bilthoven, The Netherlands; mvwillige@outlook.com (M.E.v.W.); hm.gotz@Rotterdam.nl (H.M.G.); JvanBergen@soaaids.nl (J.E.A.M.v.B.); birgit.van.benthem@rivm.nl (B.H.B.v.B.); 2 Laboratory of Immunogenetics, Department Medical Microbiology and Infection Control, Location VU University Medical Center, Amsterdam University Medical Centre (UMC), De Boelelaan 1108, 1081 HZ Amsterdam, The Netherlands; j.pleijster@amsterdamumc.nl (J.P.); samorretravel@yahoo.co.uk (S.A.M.); 3 Institute for Public Health Genomics (IPHG), Department of Genetics and Cell Biology, Research School GROW (School for Oncology & Developmental Biology), Faculty of Health, Medicine & Life Sciences, University of Maastricht, Universiteitssingel 40, 6229 ET Maastricht, The Netherlands; j.land@maastrichtuniversity.nl; 4 Department Infectious Disease Control, Municipal Public Health Service Rotterdam-Rijnmond (GGD Rotterdam), Schiedamsedijk 95, 3011 EN Rotterdam, The Netherlands; 5 Department of Public Health, Erasmus MC—University Medical Center Rotterdam, Doctor Molewaterplein 40, 3015 GD Rotterdam, The Netherlands; 6 Department of General Practice, Division Clinical Methods and Public Health, location Academic Medical Center, Amsterdam University Medical Centre (UMC), Meibergdreef 9, 1105 AZ Amsterdam, The Netherlands; 7 STI AIDS Netherlands (SOA AIDS Nederland), Keizersgracht 392, 1016 GB Amsterdam, The Netherlands; 8 Department of Sexual Health, Infectious Diseases and Environmental Health, South Limburg Public Health Service (GGD South Limburg), Het Overloon 2, 6411 TE Heerlen, The Netherlands; nicole.dukers@ggdzl.nl (N.H.T.M.D.-M.); Christian.Hoebe@ggdzl.nl (C.J.P.A.H.); 9 Department of Medical Microbiology, Care and Public Health Research Institute (CAPHRI), Maastricht University Medical Centre (MUMC+), P. Debyelaan 25, 6229 HX Maastricht, The Netherlands

**Keywords:** *Chlamydia trachomatis*, antibody testing, antibodies, chlamydia IgG, prevalence

## Abstract

The asymptomatic course of *Chlamydia trachomatis* (CT) infections can result in underestimated CT lifetime prevalence. Antibody testing might improve this estimate. We assessed CT antibody positivity and predictive factors thereof in the Netherlands Chlamydia Cohort Study. Women who had ≥1 CT Nucleic Acid Amplification Test (NAAT) in the study (2008–2011) and who provided self-reported information on NAATs were tested for CT major outer membrane protein specific IgG in serum (2016). CT antibody positivity was assessed and predictive factors were identified using multivariable logistic regressions, separately for CT-positive women (≥1 positive NAAT or ≥1 self-reported positive CT test) and CT-negative women (negative by study NAAT and self-report). Of the 3,613 women studied, 833 (23.1%) were CT -positive. Among the CT-negative women, 208 (7.5%, 95% CI 6.5–8.5) tested positive for CT antibodies. This increased CT lifetime prevalence with 5.8% (95% CI 5.0–6.5). Among women with a CT-positive history, 338 (40.6%, 95% CI 38.5–44.1) tested positive. Predictive factors for antibody positivity related to lower social economic status, sexual risk behavior, multiple infections, higher body mass index, and non-smoking. CT antibody testing significantly increased the lifetime prevalence. Combining NAAT outcomes, self-reported positive tests, and antibody testing reduced misclassification in CT prevalence estimates.

## 1. Introduction

Disease monitoring and proper evaluation of *Chlamydia trachomatis* (CT) control efforts are important and require accurate estimates of current and lifetime CT prevalence [1]. This is, however, challenging given the asymptomatic nature of the CT infection. Current prevalence estimates and our understanding of CT related complications are primarily based on studies that measure current (vaginal) infections by using nucleic acid amplification tests (NAATs) [2,3]. These tests have a high sensitivity to detect CT [4]. However, CT infections are asymptomatic in up to 70% of the cases in women [5]. Therefore, a considerable part of these infections might go unnoticed and thus remain undetected [2,6]. Presumably, an underestimation of the CT lifetime prevalence is the result [6,7]. Furthermore, estimating the proportion of infected women that later experiences CT related complications is difficult [8,9].

Given these uncertainties, several studies proposed the use of CT antibody testing as an additional means to more accurately determine CT lifetime prevalence [1,3,10]. Elevated CT IgG levels in serum are a marker of a previous CT infection and can provide information on past infection [3,11]. Undetected and cleared infections can be included in lifetime prevalence estimates and improve the accuracy in either CT surveillance or in cohort studies assessing the effects of CT infections.

However, the interpretation of CT seropositivity is not straightforward because not all women with CT infections will develop specific antibodies, furthermore, there is poor insight into the course of antibody titers, during and after CT infection, and individual variation thereof [1,12,13]. Characteristics of women with and without a positive CT NAAT test who are CT seropositive are not well-established [3,14]. We wanted to gain more insight into the benefit of CT antibody testing in estimating CT lifetime prevalence. First, we aimed to assess and compare CT antibody positivity among women with a CT-positive history (i.e., NAAT-positive results or self-reported positive test results) and in women with a negative history (NAAT-negative and no self-reported positive test). Second, we aimed to identify the predictive factors of CT antibody positivity in the two groups.

## 2. Materials and Methods

### 2.1. Study Design and Population

**Figure 1:**
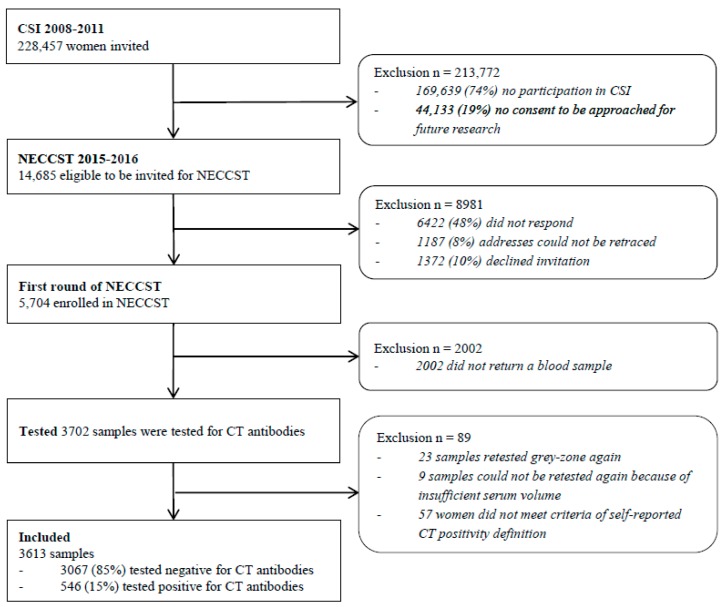
Flowchart for inclusion of participants in the present study, based on previous participation in the Chlamydia Screening Implementation (CSI) (*n* = 58,818) and Netherlands Chlamydia Cohort Study (NECCST) (*n* = 5704) projects.

We used cross-sectional data from the Netherlands Chlamydia Cohort Study (NECCST), an ongoing longitudinal cohort study of women of reproductive age in the Netherlands, prospectively followed for ≥10 years to investigate disease progression of CT [15]. NECCST is a follow-up study from the Chlamydia Screening Implementation (CSI) study, see Figure 1. In CSI, women were tested annually for CT through vaginal swabs or urine samples between 2008 and 2011 [16]. Women who participated in at least one round of CSI were recruited for NECCST in 2015–2016. All participants had ≥1 NAAT test for CT during CSI, referred to as CSI–NAAT. Between 2008–2011 (CSI) and 2015–2016 (NECCST), women completed questionnaires about previous CT tests (and results), and on demographics, health characteristics, and (sexual) risk behavior. At the start of NECCST, capillary blood samples were collected to test for CT IgG antibodies. Women were included in the present study in case there was a negative or positive CT IgG antibody result available.

### 2.2. CT Antibody Testing

Within two weeks after completing the initial NECCST questionnaire, participants received a kit at home for self-collection of capillary blood for the CT IgG antibody testing. Capillary blood was obtained via finger prick and collected in a collection tube (BD Microtainer® serum separator tube, Becton, Dickinson and Company, USA). Samples were sent by regular mail to the laboratory and were immediately processed and centrifuged, in order to collect the serum. Subsequently, serum samples were stored at −20 °C until the serological measurement was performed [17].

To test for CT antibodies, the commercially available CT IgG ELISA plus (CT IgG ELISA plus; Medac, Wedel, Germany) test was chosen because this assay is the most widely used serology test in the Netherlands, which utilizes comparability between studies. In addition, this assay is used in clinics as part of infertility diagnostics. It is a peptide-based assay and used major outer membrane protein (MOMP) as the antigen of CT. The synthetic MOMP peptide is based on all available genetic information of the CT serovar, such that they detect all CT serovars without cross reactivity to other Chlamydiae, including *Chlamydia pneumoniae* [18]. This resulted in a high specificity of 97%. Other advantages of the ELISA plus are the high-throughput and quantitative outcomes [18]. Sensitivity varied between 71–93% in comparison to the micro-immunofluorescence (MIF) or other serological tests, and between 30–77% compared to the NAAT-positive women, depending on time since infection [11,18,19]. The assay was performed according to the manufacturer’s instructions to test for the presence of CT IgG antibodies.

Quantitative serological outcomes were classified as negative (IgG concentration <22 AU/mL), grey-zone (IgG concentration 22–28 AU/mL), or positive (IgG concentration ≥28 AU/mL). Samples with a grey-zone serological outcome were retested and the retest outcome was used. In cases where the samples tested in the grey-zone again or the samples could not be retested, the participants were excluded from this study (Figure 1, study flowchart).

### 2.3. Nucleic Acid Amplification-Based Diagnostics

During the annual CSI, CT tests were performed in self-collected vaginal swabs or urine samples. These samples were tested by NAATs, which are considered to be the preferred method for diagnosis of CT infections [4]. In CSI, there was a minimum of one and a maximum of four NAAT outcomes for all women, depending on participation.

### 2.4. Questionnaires

Potential predictive factors were obtained by CSI questionnaires and the initial NECCST questionnaire. CSI questionnaires addressed previous STIs, demographics, and sexual risk behavior. The NECCST questionnaire contained questions related to CT infections, sexual health, sexual risk behavior, and health characteristics. In case of at least one self-reported CT infection, the year of the positive CT test and the CT-related symptoms, such as vaginal discharge, abdominal pain, or pain during intercourse or intermenstrual vaginal bleeding, were documented. We used CSI data where possible in order to shorten the recall distance and to verify data.

### 2.5. CT History

A CT-positive history was determined by positive NAAT outcomes during CSI and self-reported positive CT tests during CSI or NECCST. A self-reported test was defined as a positive CT test (NAAT) performed in an STI clinic or hospital, by a GP, or performed in samples collected at home but tested in a laboratory. A CT-negative history was defined as no positive NAAT result and no self-reported positive CT tests.

### 2.6. Potential Predictive Factors

First, demographics were taken into account. Age of participants was assessed at the time of the initial NECCST questionnaire. Migration background was classified as ‘Western’ if both parents had a Western country of birth (i.e., a country from Europe [excluding Turkey], North-America, Oceania, Indonesia, and Japan), ‘non-Western’ if at least one parent had a non-Western country of birth, and ‘unknown’ if one parent was Western whereas the country of birth of the other parent was unknown, or when country of birth of both parents was unknown. Educational level was categorized by low- and middle-educational level versus high-educational level (low/medium—no education, primary education only, lower general secondary education, and vocational education, high—higher professional education and university education). Other potential predictive factors were—the number of CT infections, number of CT tests, years since last infection until the year of the initial NECCST questionnaire (serum test), CT symptoms (yes/no), and previous gonorrhea infections. Factors related to sexual risk behavior were—the number of lifetime sex partners, condom use with a casual partner, condom use with a steady partner, and age of sexual debut. Last, the following health characteristics were considered—smoking and Body Mass Index (BMI).

### 2.7. Statistical Analysis

Prior to conducting analyses, the amount of missing data was investigated. In addition, it was determined whether the missing data were dependent on the participants’ characteristics or the outcome variable. Descriptive analyses were performed and characteristics of the total study population are presented. To compare characteristics between women with a positive and negative chlamydia history, the chi-squared test, *t*-tests, and the Wilcoxon rank-sum test were used.

CT antibody status was described and compared by a CT-negative history and a CT-positive history, using the chi-squared tests. The latter was further divided between CSI-NAAT positive women and women with self-reported positive CT tests only. The additional value of the CT antibody testing for estimating CT lifetime prevalence in combination with CT-NAAT and self-reported positive tests was calculated. Differences in CT lifetime prevalence’s were tested with the McNemar test.

To examine predictive factors of CT antibody positivity, multivariable logistic regression analyses were performed. The analyses were stratified by CT history (a negative history or a positive history). Univariable analyses tested associations between potential predictive factors and CT IgG antibody presence. *p*-values < 0.10 were used as cut-off for inclusion in the multivariable model. In the multivariable model, a backward stepwise selection was used in order to manually remove factors that were not significantly (*p* ≥ 0.05) associated with CT antibody positivity anymore. Results of univariable and multivariable analyses were presented as odds ratios (ORs) and adjusted odds ratios (aORs) with 95% confidence intervals (CIs). Statistical analyses were performed using STATA (Version 15.1; StataCorp, College Station, TX, USA).

### 2.8. Ethics Approval

This study was approved by the Medical Ethical Committee VU medical Center, Amsterdam the Netherlands (NL 51553.094.14/2015.903(A2019.336)), date of approval 13 October 2015.

### 2.9. Data Availability

On request, anonymized data could be provided for research related to STI, after approval by an advisory committee.

## 3. Results

In total, 3,702 blood samples were tested for CT antibodies from women who had an available NAAT result and who completed the initial NECCST questionnaire. Of the 80 grey-zone samples, 32 were excluded from analyses because of insufficient serum volume for retesting or a second grey-zone test outcome. Additionally, 57 women were excluded because they did not meet the criteria for CT history positivity resulting in 3,613 women being included in the analyses; see Figure 1. The amount of missing data was 0.5%. Missing values did not depend on participant characteristics and were assumed to be at random.

### 3.1. Participant Characteristics

Table 1 summarizes the main characteristics of all participants, *n* = 3,613. In total, 2,780 (76.9%) participants had a CT-negative history and 833 (23.1%) participants had a CT-positive history. Of participants with a CT-positive history, 206 (24.7%) had ≥1 positive CSI–NAAT result and 627 (75.3%) were based on self-reported positive CT tests only. Participants predominantly had a Western migration background (83.4%), were often highly educated (81.6%), and the mean age was 31.2 (SD 3.8, range 21–38) years in 2015/2016 at the time of CT IgG antibody test.

### 3.2. CT Antibody Status

Table 2 presents CT antibody status stratified by CT history, with a further distinction between participants with at least one CSI–NAAT positive result and participants with only self-reported positive CT tests. Of the 2,780 participants with a CT-negative history, 208, i.e., 7.5% (95% CI 6.5–8.5) tested positive for CT IgG, and among the 833 participants with a CT-positive history, 338, 40.6% (95% CI 37.2–43.9) tested positive for CT IgG (*p* < 0.001). CSI–NAAT positives were more often (*n* = 98, 47.6%, 95% CI 40.6–54.6) CT-IgG-positive, compared to women with only self-reported positive tests (*n* = 240, 38.3%, 95% CI 34.5–42.1), *p* = 0.018.

Including outcomes of the CT antibody tests in the CT history definition (i.e., combining CSI–NAAT positive tests (*n* = 206), only the self-reported positive tests (*n* = 627), and only the CT antibody positives (*n* = 208) increased the lifetime CT prevalence from 23.1% (95% CI 21.7–24.5) to 28.8% (95% CI 27.3–30.3). This was a significant increase of 5.8% (95% CI 5.0–6.5), *p* < 0.001.

### 3.3. Predictors of Having CT Antibodies

Table 3 shows univariable associations with CT antibodies and predictive factors of CT antibody positivity, identified by multivariable analyses. For women with a CT-positive history, multivariable analysis indicated that only the following factors remains associated with CT antibody positivity: unknown migration background, higher number of CT infections, and non-smoking.

For women with a CT-negative history, multivariable analysis demonstrated that the predictive factors of CT antibody positivity were non-Western or unknown migration background, low/middle-educational level, higher number of lifetime sexual partners, younger age at first intercourse, and BMI > 30.

## 4. Discussion

In this cohort of women of reproductive age, eight percent of women who previously tested negative for CT by NAAT, tested positive for CT IgG antibodies. This increased the CT lifetime prevalence estimate with six percent. Among women who previously tested positive for CT by NAAT, 40% tested positive for the CT IgG antibodies. In women with a CT-positive history (CSI–NAAT positive or self-reported positive CT tests), the predictive factors of CT antibody positivity were an unknown migration background and multiple CT infections, while occasional smoking (compared to current smoking) had a preventive effect. In women with a CT-negative history, predictive factors of previous unnoticed infections were a non-Western and unknown migration background, low- or middle-educational level, higher number of lifetime sexual partners, younger age at first sexual intercourse, and obesity.

CT IgG antibody testing identified 208 unnoticed CT infections among 2,780 women; infections that otherwise would have been missed. Therefore, in this population of women with a mean age of 31 (SD 4), lifetime CT prevalence increased by six percent from 23% (i.e., CSI–NAAT positive or self-reported positive tests) to 29%, including CT antibody testing. Although an increase of six percent suggests only a minor increase, the 208 additional CT history positives accounted for twenty percent (208/1,041) of all CT history positives. This was similar for CSI–NAAT positives that also accounted for 20% (206/1,041) of CT history positives, whereas the contribution from self-reported positive CT tests was 60%. Focusing only on NAAT and self-reported tests has its limitations. Although NAATs are highly sensitive and specific [4], only current CT infections can be measured. Median number of NAAT tests per participant in CSI was two. Including only NAAT outcomes for the CT lifetime prevalence would result in a large underestimation. Self-reported positive tests contributed the most in CT lifetime prevalence estimates, but might have induced recall bias and resulted in misclassifications in CT history. This was demonstrated in the difference in CT antibodies among women with a CSI–NAAT positive result (48% CT IgG positive) and women who only had a self-reported positive test (38% CT IgG positive). Therefore, combining NAAT outcomes, self-reported positive test outcomes, and CT antibody test outcomes results in more robust prevalence estimates. This would not erase misclassification but would reduce it. Consequently, estimation of the proportion of women who experiences CT-related complications, for example in cohort studies, would be more precise by including IgG antibody testing because there would be less misclassification in CT status [1,20].

Nevertheless, among women with a positive NAAT, less than half tested positive for CT IgG antibodies. Therefore, the proportion of women with past CT infections is presumably higher. Among CT- positive history women, 60% of infections were missed while using antibody testing. Assuming a similar test sensitivity in both the CT- positive and CT-negative group, and by applying this percentage missed to the CT- negative group, one could hypothesize that true CT (sero-) positivity is around 20% instead of 8% in the CT- negative group. However, sensitivity might differ between these two groups as in the CT- positive group, 51% of women reported symptoms and 24% of women had multiple CT infections, which could have caused higher antibody responses [11,21].

In participants with a CT-positive history, an unknown migration background and multiple CT infections were strongly associated with CT antibody positivity. This provides additional evidence that repeated exposure to CT infections is an important predictor of CT antibody positivity [11,22]. Occasional smoking compared to non-smoking was demonstrated to be a condition that decreases the likelihood of having CT IgG antibodies. We only found this association among women who previously tested positive for CT. It is, therefore, not a risk or protective factor for acquiring a CT infection, but specifically in having CT IgG antibodies following CT infection. The association might be explained by alterations in the immune response, such as an accelerated decrease of antibodies over time or weaker antibody responses. It is known that smoking can downregulate the immune system [23]. However, we did not find a significant difference with current smokers as compared to non-smokers.

In participants with a CT-negative history, predictive factors for antibody positivity could be interpreted as predictive factors for women with unnoticed infections next to predictors for seroconversion. A non-Western or unknown migration background and low/middle-educational level were predictive for antibody positivity [24,25] and both characteristics related to a lower socioeconomic status (SES), which was associated with increased sexual risk behavior [26]. Additionally, previous research found higher CT antibody responses among African-Americans compared to Caucasians [13]. A positive association was found between a high number of lifetime sexual partners and CT antibody positivity, although this association was less strong than demonstrated in a previous study [10]. Age at time of antibody test was not a risk factor for having antibodies, probably because the majority of women in the cohort were all around thirty years of age. However, younger age at first sexual intercourse also increased the risk of being CT antibody positive and was consistent with previous research [10,27]. Young age is a consistent risk factor for CT acquisition, which could suggest an acquired and protective immune response [28]. Moreover, women with obesity had an increased risk of being CT antibody positive. We do not have a clear explanation for this finding. Although obesity is a condition that affects those with a low SES more often [29], the association remained after correcting for education level and migration status in the multivariable model. This suggests that a lower SES is not an explanation for this finding. On the other hand, we cannot completely rule out residual confounding. It might be that BMI is not completely explained by the variables we have in the dataset and that BMI is still linked to a lower SES and, therefore, poses a higher risk for acquiring CT infection and not specifically CT IgG antibodies. Especially because obesity is associated with impaired immunity and reduced immunocompetence [30], we would expect to find a decreased risk for CT antibodies instead of an increased risk. It would be interesting to study a possible association between CT infection or presence of CT antibodies and BMI in other cohorts. Last, participants with a CT-negative history and a positive CT antibody test presumably did not receive treatment for the infection. This IgG test does not give any information on whether the infection was past or current. Hence, treatment is not indicated, which is in line with national and international guidelines [31].

Several limitations should be noted. The antibody test specificity (although high—97%) could, in combination with the low seroprevalence in the CT- negative group, result in a considerable number of false positives. However, the specificity issues reported in general in the past based on the potential cross reactivity with *Chlamydia pneumoniae*, *Chlamydia psittaci*, and Acinetobacter were not applicable in the current peptide-based ELISA from Medac in which a small peptide from the MOMP protein was selected with no cross reactivity with *C. pneumoniae*, in contrast to either lipopolysaccharide-based ELISAs or total MOMP-based ones [32,33,34]. Furthermore, there was the possibility of selection bias. The overall CT antibody positivity rate of 16% found in this study was higher than that found in a population-based seroprevalence study among 25–39-year old women, which was previously conducted in the Netherlands; a seroprevalence of 9% in 2007 was found [14]. A possible explanation might be that NECCST participants were at higher risk for CT when compared to the general population who participated in the population-based study. This was also seen in the screening trial. CSI participants had a higher CT positivity rate compared to the estimated population prevalence and had higher behavioral risk factors compared to people not participating in the CSI, which suggest a self-selection for screening, based on personal risk perception [16,35]. This might influence the generalizability of the results. The 8% antibody positives in the CT-negative group could be an overestimation when compared to the general population.

To gain more insight into the proportion of women with unnoticed previous CT infections and thereby improve the prevalence estimates even more, further research should be conducted. For example, assays based on different CT antigens such as the Pgp3 in combination with MOMP-based assays or assays using a range of CT antigens to determine the most reactive CT antigens might increase insight [12,36,37]. Additionally, the effect of time on antibody levels requires further study. In our multivariable analyses, time measured in years, since last infection was not a predictive factor for CT antibody positivity in multivariable analysis. According to a study by Horner et al. [11], antibodies were detected in only 38% of participants from genitourinary medicine clinics six months after infection, as compared to 77% at time of infection, using the ELISA plus from Medac [19,21]. This suggests that the antibody levels decreased within the first months after infection, but no significant further reduction happened after six months. This was comparable with a recent study in which the same assay was used, antibodies were measured within three months and three to ten years after infection; seroprevalence was 66% and 35%, respectively [22].

In conclusion, we demonstrated that CT IgG antibody testing identified women with unnoticed CT infections among women previously tested negative for CT by NAAT. Although not all women tested positive for CT antibodies despite a previous CT infection, the extra infections found with antibody testing significantly increased the CT lifetime prevalence estimates. Consequently, this improved the estimates on proportions of women who experienced CT-related complications. In estimating the CT lifetime prevalence, we recommended CT antibody testing in combination with previous chlamydia test outcomes.

## Figures and Tables

**Table 1:** Main characteristics of the study population.

	Total *n* = 3613	CT-Negative History ^†^ *n* = 2780 (76.9%)	CT-Positive History ^‡^ *n* = 833 (23.1%)	*p*-Value
**Demographic characteristics**				
Age				0.089
Q1: 21–28 years	923 (25.6)	703 (25.3)	220 (26.4)	
Q2: 29–31 years	954 (26.4)	721 (25.9)	233 (28.0)	
Q3: 32–34 years	899 (24.9)	685 (24.6)	214 (25.7)	
Q4: 35–38 years	837 (23.2)	671 (24.1)	166 (19.9)	
Migration background (*n* (%))				<0.001
Western	3017 (83.5)	2391 (86.0)	626 (75.2)	
Non-western	446 (12.3)	277 (10.0)	169 (20.3)	
Unknown	150 (4.2)	112 (4.0)	38 (4.6)	
Educational level ^# a^ (n (%))				<0.001
Low and middle	661 (18.3)	439 (15.8)	222 (26.7)	
High	2949 (81.7)	2339 (84.2)	610 (73.3)	
**Infection characteristics**				
Number of CT infections (*n* (%))				
0 infections	2780 (76.5)	2,780 (100.0)		
1	631 (17.5)		631 (75.8)	
2	166 (4.6)		166 (19.9)	
≥3	36 (1.0)		36 (4.3)	
Number of CT tests (*n* (%))				<0.001
<2 tests	609 (16.9)	576 (20.7)	33 (4.0)	
2	994 (27.5)	852 (30.7)	142 (17.1)	
3	968 (26.8)	748 (26.9)	220 (26.4)	
>3	1042 (28.8)	604 (21.7)	438 (52.6)	
Years since last CT infection ^b^ (median IQR)	6.0 (4.0–9.0)		6.0 (4.0–9.0)	
CT symptoms ^c^^$^ (*n* (%))				
No symptoms	408 (49.0)		408 (49.0)	
Symptoms	425 (51.0)		425 (51.0)	
**Sexual behavior**				
Condom use with steady partner ^d^ (*n* (%))				0.001
Always, mostly	309 (8.6)	260 (9.4)	49 (5.9)	
Sometimes	583 (16.2)	439 (15.9)	144 (17.3)	
Never/not often	1000 (27.8)	734 (26.5)	266 (32.0)	
At beginning of relationship	1665 (46.2)	1301 (47.0)	364 (43.8)	
No steady partners	45 (1.3)	36 (1.3)	9 (1.1)	
Condom use with casual partner ^e^ (*n* (%))				<0.001
Always, mostly	1662 (46.1)	1313 (47.4)	349 (42.0)	
Sometimes	1131 (31.4)	762 (27.5)	369 (44.4)	
Never/not often	217 (6.0)	165 (6.0)	52 (6.3)	
No casual partners	592 (16.4)	530 (19.1)	62 (7.5)	
Number of lifetime sexual partners (*n* (%))				<0.001
<6 partners	1163 (32.2)	1034 (37.2)	129 (15.5)	
6–12	1243 (34.4)	956 (34.4)	287 (34.5)	
>12	1207 (33.4)	790 (28.4)	417 (50.1)	
Age of first intercourse (*n* (%))				<0.001
≤15 years	926 (25.6)	647 (23.3)	279 (33.5)	
16–17	1419 (39.3)	1076 (38.7)	343 (41.2)	
≥18	1268 (35.1)	1057 (38.0)	211 (25.3)	
Self-reported gonorrhea infection (*n* (%))				<0.001
Never	3555 (98.4)	2758 (99.2)	797 (95.7)	
At least one	58 (1.6)	22 (0.8)	36 (4.3)	
**Lifestyle characteristics**				
Smoking status (*n* (%))				<0.001
Never	1455 (40.3)	1205 (43.3)	250 (30.0)	
Occasional	1813 (50.2)	1351 (48.6)	462 (55.5)	
Daily	345 (9.6)	224 (8.1)	121 (14.5)	
BMI ^f^ (median (IQR))	22.5 (20.8–24.8)	22.5 (20.8–24.6)	22.7 (20.8–25.3)	0.117

Data are medians ± inter quartile ranges (IQR) or numbers and percentages. Percentages might not add up to a total of 100 as values were rounded-up. CT = *Chlamydia trachomatis*, Q = quartile, BMI = Body Mass Index. ^†^ CT-negative history was defined as: no positive Nucleic Acid Amplification Test (NAAT) result during the Chlamydia Screening Implementation study (CSI) [16] and no self-reported positive CT test. ^‡^ CT-positive history was defined as—at least one NAAT positive result during CSI or self-reported positive CT tests. ^o^ Migration background was classified as ‘Western’ if both parents had a Western country of birth (i.e., a country from Europe [excluding Turkey], North-America, Oceania, Indonesia, and Japan), ‘non-Western’ if at least one parent had a non-Western country of birth, and ‘unknown’ if one parent was Western whereas the country of birth of the other parent was unknown or when country of birth of both parents was unknown. ^#^ Educational level was categorized by low- and middle-educational level versus high-educational level (low/medium—no education, primary education only, lower general secondary education, and vocational education, high—higher professional education and university education). ^$^ CT symptoms included—vaginal discharge, abdominal pain or pain during intercourse or intermenstrual vaginal bleeding. ^a^ missing 3. ^b^ missing 3: based on women (*n* = 833) with a CT-positive history. ^c^ based on women (*n* = 833) with a CT-positive history. ^d^ missing 11; ^e^ missing 11; ^f^ missing 4.

**Table 2:** CT IgG antibody status in women with a CT-positive history and a CT-negative history.

		CT IgG Positive	Total	*p* Value
CT-negative history ^†^		208 (7.5%)	2780 (100%)	<0.001 *
CT-positive history ^‡^		338 (40.6%)	833 (100%)	
	NAAT positive in CSI	98 (47.6%)		0.018 **
	Self-reported positive	240 (38.3%)		
Total		546 (15.1%)	3613 (100%)	

CT—*Chlamydia trachomatis*, IgG—Immunoglobulin G, NAAT—Nucleic Acid Amplification Test, CSI—Chlamydia Screening Implementation study. ^†^ CT-negative history was defined as: no positive NAAT result during the CSI [16] and no self-reported positive CT test. ^‡^ CT-positive history was defined as: at least one NAAT positive result during CSI or self-reported positive CT test. * *p* value of the difference in CT IgG positivity between CT history negative and CT history positive women. ** *p*-value of the difference in CT IgG positivity between CSI–NAAT positives and self-reported positive tests only.

**Table 3:** Univariable and multivariable analyses of *Chlamydia trachomatis* (CT) IgG antibody status stratified for women with a CT-negative and a CT-positive history.

	Positive CT IgG Antibody Status			
	CT-Negative History ^†^		CT-Positive History ^‡^	
CT IgG Antibody Positivity n/N	207/2768		337/830	
	Crude	Adjusted	Crude	Adjusted
**Variable**	OR (95% CI)	aOR (95% CI)	OR (95% CI)	aOR (95% CI)
Age				
Q1: 21–28 years *	1		1	
Q2: 29–31 years	0.95 (0.63–1.44)		1.11 (0.76–1.63)	
Q3: 32–34 years	1.05 (0.70–1.58)		1.09 (0.74–1.60)	
Q4: 35–38 years	1.33 (0.90–1.97)		1.27 (0.84–1.91)	
Migration background ^o^				
Western *	1	1	1	1
Non-Western	**2.19 (1.49–3.21)**	**2.22 (1.49–3.29)**	**1.47 (1.04–2.06)**	1.38 (0.97–1.96)
Unknown	**2.03 (1.13–3.64)**	**2.10 (1.16–3.83)**	1.81 (0.94–3.49)	**1.98 (1.00–3.91)**
Educational level ^# a^				
Low and middle *	1	1	1	
High	**0.50 (0.36–0.70)**	**0.58 (0.41–0.82)**	0.84 (0.61–1.14)	
Number of CT infections				
1 *			1	1
≥2			**2.35 (1.71–3.25)**	**2.28 (1.64–3.17)**
Number of CT tests				
<2 tests *	1		1	
2	0.77 (0.51–1.17)		1.06 (0.49–2.30)	
3	1.07 (0.72–1.61)		0.93 (0.44–1.97)	
>3	1.14 (0.75–1.73)		1.11 (0.54–2.30)	
Years since last CT infection ^b^				
<2 years *			1	
2–5			0.73 (0.44–1.21)	
>5			**0.65 (0.41–1.03)**	
CT symptoms ^$ c^				
No symptoms *			1	
Symptoms			1.14 (0.86–1.50)	
Condom use with steady partner ^d^				
Always, mostly *	1		1	
Sometimes	1.10 (0.63–1.95)		1.45 (0.75–2.80)	
Never/not often	0.84 (0.49–1.44)		0.96 (0.52–1.79)	
At beginning of relationship	0.99 (0.60–1.63)		0.87 (0.47–1.59)	
No steady partners	1.10 (0.31–3.87)		1.16 (0.28–4.86)	
Condom use with casual partner ^e^				
Always, mostly *	1		1	
Sometimes	**1.32 (0.96–1.82)**		0.99 (0.73–1.33)	
Never/not often	0.65 (0.31–1.35)		1.00 (0.55–1.81)	
No casual partners	0.79 (0.52–1.20)		1.21 (0.71–2.09)	
Number of lifetime sexual partners				
<6 partners *	**1**	**1**	1	
6–12	**1.68 (1.19–2.39)**	**1.76 (1.22–2.53)**	0.90 (0.58–1.37)	
>12	**1.64 (1.14–2.37)**	**1.81 (1.23–2.68)**	1.17 (0.78–1.75)	
Age at first intercourse				
≤15 years *	**1**	1	1	
16–17	**0.70 (0.50–0.98)**	0.75 (0.53–1.07)	0.83 (0.60–1.15)	
≥18	**0.56 (0.39–0.80)**	**0.62 (0.42–0.90)**	0.96 (0.67–1.38)	
Self-reported gonorrhea infection				
Never *	**1**		**1**	
At least one	**2.78 (0.93–8.30)**		**2.11 (1.08–4.17)**	
Smoking status				
Never *	1		1	1
Occasional	1.07 (0.79–1.45)		**0.73 (0.54–1.00)**	**0.70 (0.51–0.97)**
Daily	**1.96 (1.24–3.08)**		1.36 (0.88–2.10)	1.17 (0.75–1.84)
BMI ^f^				
Healthy weight (18.5–25) *	1	1	1	
Underweight (<18.5)	0.76 (0.27–2.11)	0.76 (0.27–2.14)	0.58 (0.24–1.40)	
Overweight (25–30)	1.15 (0.78–1.69)	1.09 (0.73–1.62)	1.19 (0.83–1.69)	
Obese (≥30)	**2.33 (1.49–3.65)**	**1.81 (1.13–2.91)**	**1.81 (1.10–2.97)**	

OR—odds ratio, aOR—adjusted odds ratio, CI—confidence interval, CT—*Chlamydia trachomatis*, IgG—Immunoglobulin G, Q—quartile, BMI—Body Mass Index. Factors associated (*p* < 0.10) in the univariable analyses were included in the multivariable analysis. Significant (*p* < 0.05) results are presented in bold. ^*^ Reference category. ^†^ CT-Negative history was defined as ‘no positive Nucleic Acid Amplification Test (NAAT) result during the Chlamydia Screening Implementation study (CSI) [16] and no self-reported positive CT tests’. ^‡^ CT-Positive history was defined as ‘at least one NAAT positive result during CSI or self-reported positive CT test’. ^o^ Migration background was classified as ‘Western’ if both parents had a Western country of birth (i.e., a country from Europe [excluding Turkey], North-America, Oceania, Indonesia, and Japan), ‘non-Western’ if at least one parent had a non-Western country of birth, and ‘unknown’ if one parent was Western whereas the country of birth of the other parent was unknown or when country of birth of both parents was unknown. ^#^ Educational level was categorized by low- and middle-educational level versus high-educational (low/medium—no education, primary education only, lower general secondary education, and vocational education, high—higher professional education and university education). ^$^ CT symptoms included—vaginal discharge, abdominal pain or pain during intercourse or intermenstrual vaginal bleeding. ^a^ missing 3. ^b^ missing 3—based on women (*n* = 833) with a CT-positive history. ^c^ based on women (*n* = 833) with a CT-positive history. ^d^ missing 11. ^e^ missing 11. ^f^ missing 4.
